# DNA damage response and cell cycle regulation in bacteria: a twist around the paradigm

**DOI:** 10.3389/fmicb.2024.1389074

**Published:** 2024-03-28

**Authors:** Hari Sharan Misra, Yogendra Singh Rajpurohit

**Affiliations:** ^1^School of Sciences, Gandhi Institute of Technology and Management (GITAM), Visakhapatnam, India; ^2^Molecular Biology Division, Bhabha Atomic Research Centre, Mumbai, India; ^3^Life Sciences, Homi Bhabha National Institute (DAE Deemed to be University), Mumbai, India

**Keywords:** bacteria, *Deinococcus*, DNA damage response, cell cycle regulation, phosphorylation, Ser/Thr protein kinase, radioresistance

## Abstract

The co-protease activity in the RecA-ssDNA complex cleaves the autorepressor LexA, resulting in the derepression of a large number of genes under LexA control. This process is called the SOS response, and genes that are expressed in response to DNA damage are called SOS genes. The proteins encoded by the SOS genes are involved in both DNA repair and maintaining the functions of crucial cell division proteins (e.g., FtsZ) under check until the damaged DNA is presumably repaired. This mechanism of SOS response is the only known mechanism of DNA damage response and cell cycle regulation in bacteria. However, there are bacteria that do not obey this rule of DNA damage response and cell cycle regulation, yet they respond to DNA damage, repair it, and survive. That means such bacteria would have some alternate mechanism(s) of DNA damage response and cell cycle regulation beyond the canonical pathway of the SOS response. In this study, we present the perspectives that bacteria may have other mechanisms of DNA damage response and cell cycle regulation mediated by bacterial eukaryotic type Ser/Thr protein kinases as an alternate to the canonical SOS response and herewith elaborate on them with a well-studied example in the radioresistant bacterium *Deinococcus radiodurans*.

## Introduction

*Escherichia coli* exposed to DNA-damaging agents triggers a series of molecular steps, including the interaction of recombinase A with damaged DNA sites in the genome (Radman, 1975). RecA binding to a single-strand region of the genome expresses its co-protease activity, which cleaves both LexA, an autorepressor of SOS regulon, and UmuD, a translesion DNA polymerase in *E. coli* (Little and Mount, 1982; Walker, 1984). The expression of more than 100 genes is altered in response to DNA damage in *E. coli*, possibly due to the inactivation of LexA (Fernandez de Henestrosa et al., 2000; Courcelle et al., 2001; Simmons et al., 2008). The majority of them are known to be DNA metabolic proteins that take part in DNA repair and other functions required to maintain genome integrity. This SOS repair mechanism involves error-prone DNA polymerases and has thus been used in the isolation of UV-induced mutants in bacteria (Witkin, 1989). Since the continuation of cell division until the damaged sites are repaired would become detrimental to the fitness of bacteria, some of these SOS proteins, e.g., SulA, help in arresting cell growth by attenuating FtsZ (the master protein of cell division) functions (Mukherjee et al., 1998; Chen et al., 2012). It is understood that FtsZ attenuation by SulA is brought down by the action of specific proteases that would cleave SulA and resume FtsZ activity, as well as by *de novo* synthesis of FtsZ. The cellular and molecular events imbibed post-DNA damage and arrest of cell division have been collectively termed DNA damage response and cell cycle regulation (SOS response) in *E. coli*. Thereafter, the RecA/LexA-type canonical SOS response that is observed in *E. coli* becomes, in general, the synonym of DNA damage response and cell cycle regulation in bacteria. Interestingly, all the studies carried out on the mechanisms underlying DNA damage response and cell cycle regulation in bacteria have concentrated around the paradigm of the RecA/LexA-type canonical SOS response. However, there are a large number of bacteria, including *Deinococcus radiodurans*, that do not contain some of the key components of LexA/RecA-type SOS mechanisms and thus do not depend upon this canonical SOS response as the mechanism(s) of DNA damage response and cell cycle regulation.

## *Deinococcus radiodurans* lacks the canonical pathway of DNA damage response

*D. radiodurans* is known for its extraordinary resistance to both physical and chemical DNA-damaging agents, including γ radiation (Slade and Radman, 2011; Misra et al., 2013). This bacterium can withstand a dose of γ radiation that produces approximately 200 double-strand breaks (DSBs) and 3,000 single-strand breaks (Cox and Battista, 2005; Zahradka et al., 2006). The genome of this bacterium encodes RecA and 2 orthologs of LexA; LexA1 (DR_A0344) and LexA2 (DR_0074; White et al., 1999). Further, the RecA of *D. radiodurans* prefers dsDNA for binding as compared to ssDNA (Ngo et al., 2013). Molecular studies on the involvement of *E. coli*-type co-protease activity in the expression of Deinococcus RecA and its functional interaction with LexA orthologs were carried out independently (Narumi et al., 2001; Bonacossa de Almeida et al., 2002; Sheng et al., 2004; Jolivet et al., 2006; Satoh et al., 2006). These findings suggest that though levels of *recA* transcript increase in response to DNA damage, this was found to be independent of the proteolytic cleavage of LexA, and/or the inactivation of *lexA* genes. Interestingly, the genome of this bacterium contains LexA boxes, and these were mapped upstream to the coding sequence of several proteins (Khan et al., 2008). Furthermore, the homologs of UmuD, the translesion DNA polymerase in *E. coli*, were not found in the genome of *D. radiodurans* (White et al., 1999). Based on this, the *E. coli*-type DNA damage response and cell cycle regulation have been ruled out in *D. radiodurans* through independent studies. This would not have been surprising because the canonical SOS response is known to engage an error-prone DNA repair pathway, and this bacterium is suggested to have a very high fidelity in the DNA repair process. Furthermore, this bacterium was found to adjust both its proteome and transcriptome upon γ radiation exposure (Liu et al., 2003; Joshi et al., 2004; Tanaka et al., 2004), arguing in favor of some mechanism that would help this bacterium to respond to the γ radiation effects on genome functions and protein homeostasis.

## Regulation of gene expression in response to DNA damage

Transcriptome analysis of *D. radiodurans* cells exposed to γ radiation and desiccation produced DSBs and showed differential gene expression (Liu et al., 2003; Tanaka et al., 2004). Mechanisms underlying differential regulation of gene expression upon γ radiation exposure are not fully understood. Earlier studies have reported that IrrE (also called PprI) acts as a master regulator of *recA* expression in this bacterium (Earl et al., 2002). Subsequently, it was found out that PprI works through DdrO, where it binds to a *cis element* named radiation desiccation response motifs (RDRMs) and keeps all genes containing RDRMs repressed (Makarova et al., 2007; Narasimha and Basu, 2021). PprI is characterized as a metalloprotease that cleaves DdrO and results in the derepression of DdrO-repressed genes (Devigne et al., 2015; Wang et al., 2015; Blanchard et al., 2017). CHIP-seq and RNA-seq analyses as a function of DdrO showed that a large number of DNA metabolism genes, including *ddrA*, *ddrB*, *ddrC*, *ddrD*, *gyrA*, *gyrB*, *ssb*, *mutS,* and *ruvB*, are under the control of DdrO repression (Eugénie et al., 2021). Independently, it has also been found that DNA damage responsiveness of gene expression in response to γ radiation is under the regulation of guanine quadruplex (G4) DNA structure dynamics (Kota et al., 2015). The genome of this bacterium is GC-rich and contains a very high density of guanine repeats, forming putative G4 DNA motifs (Beaume et al., 2013). It has been shown that G4 DNA structure plays crucial roles in genome functions in response to γ radiation-mediated DNA damage (Mishra et al., 2019). However, all the DNA damage-responsive genes do not confer G4 DNA motifs, neither in their regulatory region nor in their coding sequences. Thus, these mechanisms though explained the regulation of expression of certain genes, they were not found to be analogous to the *E. coli* type canonical DNA damage response and cell cycle regulation in this bacterium.

## Cross-talk of oxidative stress response with DNA damage repair in *Deinococcus radiodurans*

Reactive nitrogen species (RNS) and reactive oxygen species (ROS) are constantly formed as side products of biological reactions. However, their levels increase in response to environmental stressors, including ionizing radiation. These free radicals contribute to a few less understood mechanisms of cellular signaling, mostly through the oxidative damage of biomolecules. The *D. radiodurans* genome annotates a gene (*pqqE*) encoding a putative pyrroloquinoline quinone (PQQ) synthase (White et al., 1999), the key enzyme of the PQQ biosynthetic pathway in bacteria (Adachi et al., 1992). *pqqE*-expressing *E. coli* showed protection against the photodynamic effect of Rose Bengal (Khairnar et al., 2003). PQQ by itself was also characterized as an antioxidant and radioprotector *in vitro* (Misra et al., 2004). The *pqqE* deletion mutant of *D. radiodurans* showed the absence of PQQ, and these cells showed the loss of γ radiation resistance and DSB repair, suggesting PQQ role(s) in radioresistance beyond its antioxidant nature (Rajpurohit et al., 2008). Historically, PQQ was known as a coenzyme for some dehydrogenases, including gluconate dehydrogenase and methanol dehydrogenase, and the beta-propeller motifs in these dehydrogenases were mapped to be the site of PQQ interaction, termed the PQQ interaction motif (PQIM) (Adachi et al., 1992; Anthony and Ghosh, 1998). The *D. radiodurans* genome contains five ORFs encoding proteins, such as DR_2518, DR_1769, DR_0503, DR_0766, and DR_C0015, which have PQIM. Albeit in varying numbers and at varying positions, in their primary structures. These genes were deleted from the genome, and the deletion mutants were checked for γ radiation response. Except for the deletion mutant of DR_2518 (Δ2518), the other deletion mutants did not affect the γ radiation response of wild-type cells. The Δ2518 cells showed approximately 3-log cycle drops at 6.5 kGy γ radiation, a dose at which the wild-type cells show more than 99% cell survival, and an impaired DSB assembly (Rajpurohit and Misra, 2010). DR2518 was subsequently characterized for its interaction with PQQ, and its autokinase and transkinase activities were stimulated by both PQQ and DNA ends *in vitro* (Rajpurohit and Misra, 2010; Sharma et al., 2022). Furthermore, the expression of *pqqE* and DR_2518 ORF and the kinase activity of DR2518 were stimulated in response to γ radiation *in vivo* (Liu et al., 2003; Rajpurohit and Misra, 2010). Thus, DR2518 was summarized as a radiation-responsive Ser/Thr quinoprotein kinase and designated as RqkA.

## *Deinococcus radiodurans* encodes putative substrate for RqkA

Based on *in silico* analysis of the potential Ser/Thr phosphorylation sites in the substrates for eSTPKs, a number of deinococcal proteins are predicted as the putative substrates of RqkA (Misra et al., 2013). The notable ones include RecA, PprA, DnaA, FtsZ, FtsA, and DivIVA. Among these, PprA was identified as a pleiotropic protein having roles in radiation resistance (Narumi et al., 2004; Murakami et al., 2006). It is now characterized for its functional interaction with a large number of proteins involved in genome integrity, including RecA during DSB repair, topoisomerases, and cell division proteins (Kota and Misra, 2006; Kota et al., 2014; Rajpurohit et al., 2021). The effect of RqkA phosphorylation on the functions of some of the DNA repair and cell division proteins was further monitored. The RqkA phosphorylation of RecA, PprA, FtsZ, and DivIVA has been demonstrated, and their phosphosites have been mapped (Rajpurohit and Misra, 2013; Rajpurohit et al., 2016; Maurya et al., 2018; Sharma et al., 2020). Phospho-mutants of these proteins have been produced, and their functions have been characterized both *in vivo* and *in vitro.* Notably, the RecA and PprA phosphorylations have enhanced their functional efficiency *in vitro* and have been found helpful in supporting their functions in the radioresistance of *D. radiodurans in vivo* (Rajpurohit and Misra, 2013; Rajpurohit et al., 2016). Similarly, FtsZ phosphorylation has also affected the functional interaction of FtsZ with FtsA as well as the polymerization dynamics of FtsZ *in vitro*. Cells expressing phosphor-mimetic mutants of FtsZ behaved differently than cells expressing phosphor-ablative and wild type, indicating the arrest of FtsZ function upon phosphorylation by RqkA (Chaudhary et al., 2023a). Furthermore, DivIVA phosphorylation at the T19 site had altered its typical pattern of localization near the new septum site and completely distorted its ability to determine the plane of the next division that is normally perpendicular to the previous plane of cell division (Chaudhary et al., 2023b) The enhancement of DNA repair function with the concurrent arrest of cell division upon Ser/Thr phosphorylation by RqkA nearly mimics these processes upon DNA damage as observed in eukaryotes (Sancar et al., 2004). Thus, a possibility of RqkA role in DNA damage response and cell cycle regulation in this bacterium, nearly similar to the Ser/Thr protein kinase (STPK) in eukaryotes (eSTPK) roles in eukaryotes, could be hypothesized.

**Figure 1:**
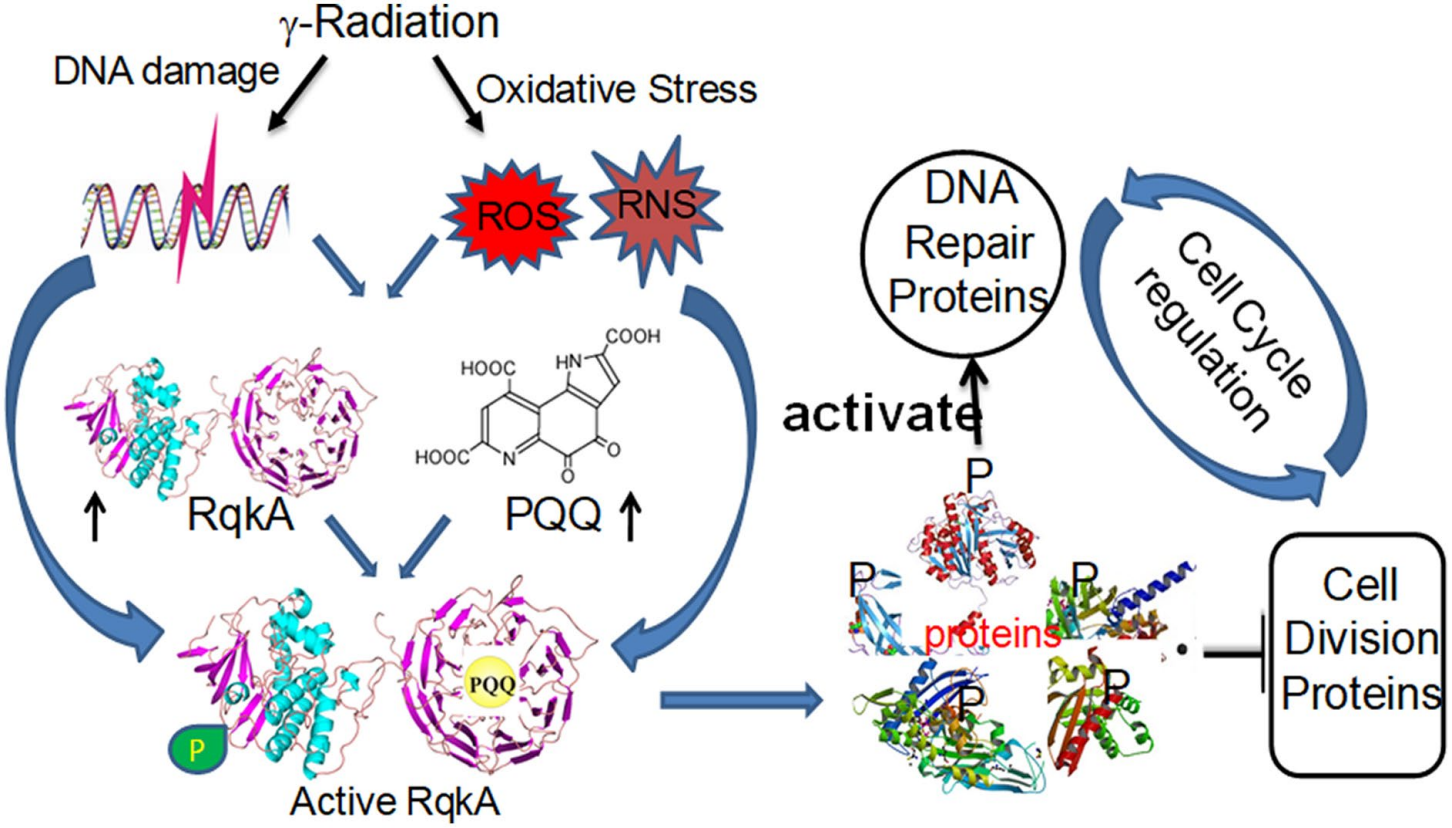
Schematic representation of eukaryotic-type γ radiation-responsive Ser/Thr quinoprotein kinase (RqkA) regulation of DNA damage response and cell cycle regulation in the radioresistant bacterium *Deinococcus radiodurans*. The γ radiation induces the synthesis of both RqkA and pyrroloquinoline quinone (PQQ). The PQQ activates the autokinase activity of RqkA, which is predicted to have a number of putative substrates in *D. radiodurans* that include DNA repair proteins such as RecA and PprA, and cell division proteins such as FtsZ. All three proteins undergo phosphorylation *in vivo,* and their phosphorylation showed a kinetic change during post-irradiation recovery. On one hand, the phosphorylation of RecA and PprA enhances their activity and is required for efficient function in the radioresistance of this bacterium. On the other hand, FtsZ phosphorylation makes it inferior, and it loses its dynamics *in vivo*.

In conclusion, DNA damage-induced cleavage of LexA by the co-protease activity of RecA is known to de-repress a large number of genes in *E. coli*. Some of these genes encode proteins that are required for the repair of damaged genomes, while others make cells stop growing by attenuating the function of the key cell division protein FtsZ, and thus have been presented as a typical model of cell cycle regulation in many bacteria. RqkA phosphorylation of both DNA repair proteins such as RecA and PprA and cell division proteins such as FtsZ and DivIVA (studied so far) and the effect of phosphorylation on their activity regulation offer an analogous model of DNA damage response and cell cycle regulation in this bacterium that seems to be nearly similar to eukaryotes (Figure 1). The roles of Ser/Thr phosphorylation in the regulation of DNA damage response and cell division in bacteria have been debated for quite some time and have been reviewed recently (Rajpurohit et al., 2022). Thus, these developments strongly suggest the possibility of the presence of STPK-based mechanisms in the regulation of DNA damage response and cell cycle in bacteria that may or may not have RecA/LexA-type canonical SOS responses, a known paradigm of DNA damage response and cell cycle regulation in bacteria.

## A potential hypothesis and future scope in research

Genome sequence analysis of a large number of bacteria did not show the presence of the components involved in the canonical LexA/RecA-type SOS response mechanism of DNA damage response and cell cycle regulation in bacteria. On the other hand, the majority of these genomes encode for signaling kinases that include eSTPKs (Misra et al., 2013). The unanswered question is how such bacteria control the cell cycle when their genomes are assaulted by genotoxic agents. The case study reported here is restricted to a bacterium and does not claim to have provided enough evidence about the existence of STPK-mediated DNA damage response and cell cycle regulation in other bacteria. It has brought forth strong evidence to suggest that Ser/Thr phosphorylation plays a very important role in the differential regulation of the activity of DNA repair and cell division proteins in *D. radiodurans*. It might allow for a meaningful debate on whether other bacteria have similar mechanisms or not. STPKs/phosphatase-mediated phosphorylation /dephosphorylation are known to regulate DNA damage response and cell cycles in higher organisms. Bacterial genome sequencing has revealed several cytogenetic features, including multipartite genome systems and ploidy, that were not believed to be the case in bacteria. Further, there are bacterial genomes that have a very high density of eSTPKs and apparently lack LexA/RecA-type SOS responses. Therefore, exploring the possibility of STPK-mediated DNA damage response and cell cycle regulation in other bacteria would be worth looking at. High-throughput omics studies (e.g., proteomics for phosphoproteome and RNA sequencing for understanding the role of phosphorylation on the regulation of gene expression) would greatly help to produce rapid, reliable, and conclusive data that may allow the microbial world to discover the new aspects of microbial physiology and biochemistry in response to stresses, very close to higher organisms.

## Data availability statement

The original contributions presented in the study are included in the article/supplementary material, further inquiries can be directed to the corresponding author.

## Author contributions

HM: Conceptualization, Formal analysis, Investigation¸ Project administration, Resources, Supervision, Validation, Writing – original draft, Writing – review & editing. YR: Conceptualization, Data curation, Formal analysis, Investigation, Methodology, Validation, Visualization, Writing – review & editing.
